# Heparin-Induced Thrombocytopenia and Portal Vein Thrombosis in Patients With Hepatocellular Carcinoma After Hepatectomy: A Case Report

**DOI:** 10.7759/cureus.79339

**Published:** 2025-02-19

**Authors:** Ryotaro Sakio, Atsushi Miki, Yasunaru Sakuma, Hideki Sasanuma, Hironori Yamaguchi

**Affiliations:** 1 Department of Surgery, Division of Gastroenterological, General and Transplant Surgery, Jichi Medical University, Shimotsuke, JPN

**Keywords:** argatroban, heparin, liver surgery, portal embolization, thrombocytopenia

## Abstract

Portal vein thrombosis and heparin-induced thrombocytopenia (HIT) caused by postoperative heparin administration is a potentially fatal disease. There have been a few cases of portal vein thrombosis and HIT developing after hepatectomy. We report a rare case of HIT and portal vein thrombosis after hepatectomy. A 79-year-old woman with liver cirrhosis and hypertension was referred to our hospital for the evaluation of the elevated tumor marker and liver tumor diagnosed by ultrasonography. In laboratory findings, alpha-fetoprotein and protein induced by vitamin K absence were elevated. Contrast-enhanced computed tomography (CT) scan findings showed that 28 mm and 17 mm diameter, round-shaped tumors enhanced heterogeneously with infiltration in the left hepatic vein and pulmonary embolization. Hepatocellular carcinoma and pulmonary embolism were diagnosed. She underwent lateral segmentectomy along with venous tumor embolus resection. Pathological findings showed that the tumor was moderate to well-differentiated adenocarcinoma invading the capsule with liver cirrhosis. Tumor embolization was suspected preoperatively to be an organizing thrombus. After surgery, she was treated with heparin for the prevention of deep venous thrombosis. At postoperative day 15, the sudden onset of the decline of platelets, the activity of antithrombin III, and the elevation of D-dimer were found. On physical examination, there were no symptoms. The 4T’s score was 7. CT scan showed a portal vein thrombosis. The antibody for HIT was elevated. HIT was diagnosed. We stopped the heparin and started the argatroban administration. The platelet level increased to the normal range, and the D-dimer level decreased. After the reduction of portal vein and pulmonary thrombosis, she was discharged from the hospital on postoperative day 28.

## Introduction

In recent years, guideline-based measures have been implemented to prevent perioperative venous thromboembolism [1]. Patients undergoing surgery for the treatment of malignant lesions (laparotomy and laparoscopy) are in a high-risk group for developing thromboembolism, and anticoagulant prophylaxis should be started between 12 h and 2 h preoperatively and continued for at least 7-10 days postoperatively with once-daily low-dose low molecular weight heparins or low-dose unfractionated heparin thrice-daily [1]. Heparin-induced thrombocytopenia (HIT) has been rarely reported among patients undergoing gastrointestinal surgery [2]. HIT is a condition where heparin induces the formation of antibodies that activate platelets, leading to both thrombocytopenia and paradoxical thrombosis. This is a rare but serious complication that can occur after surgery in patients receiving heparin. Portal vein thrombosis may be a lethal disease after surgery, challenging anticoagulation and inducing portal hypertension in the perioperative period [3]. While portal vein thrombosis is a known complication after hepatectomy, it is extremely rare for portal vein thrombosis to be caused by HIT. This report aims to explore such an unusual presentation and highlight diagnostic and therapeutic strategies.

## Case presentation

The patient is a 79-year-old female followed up with liver cirrhosis and hypertension in the clinic. The elevation of alpha-fetoprotein and a liver nodule detected by ultrasonography were pointed out in the routine examination. For further examination, she was referred to our hospital. In laboratory examination, aspartate aminotransferase (40 IU/l), alanine aminotransferase (35 IU/l), alkaline phosphatase (396 IU/l), alpha-fetoprotein (18594 ng/ml), protein-induced vitamin k absence (272 mau/ml) were increased (Table 1).

**Table 1:** Laboratory data. HIT: heparin-induced thrombocytopenia; POD: Postoperative day; WBC: White blood cell; Hgb: Hemoglobin; Hct: Hematocrit; PT: Prothrombin time; PT-INR: Prothrombin Time-International Normalized Ratio; AT-III: Antithrombin III; AST: Aspartate transferase; ALT: Alanine transaminase; AFP: Alpha-fetoprotein; PIVKA-II: protein induced by vitamin K absence-II

Parameters	Admission	Onset of HIT	POD 25	Reference range	Unite
WBC	5.0	6.6	7.2	3.5-9.1	(× 10^3^cells/μl)
Hgb	13.9	9.8	10.8	11.3-15.2	(× g/dl)
Hct	42.2	30	33.5	33.4-44.9	%
Platelets	129	46	184	13.0-36.9	(× 10^3^cells/μl)
PT	12.3	14.8	15.1	10.4-12.2	s
PT-INR	1.07	1.30	1.33	0.9-1.2	
APTT	32.2	40.5	34.4	23.1-36.3	s
PT%	98.6	66.1	54.2	80-100	%
D-dimer	0.6	23.5	2.2	0-1.5	(μg/ml)
AT-III activity		49.3	49.9	88-116	%
Albumin	4.3	2.6	3.3	3.9-5.1	g/dl
Total Bilirubin	0.93	0.70	0.66	0.4-1.50	mg/dl
Direct Bilirubin	0.19	0.14	0.11	0.06-0.23	mg/dl
AST	40	19	26	11.0-30.0	U/I
ALT	35	31	24	4.0-30.0	U/I
LDH	202	180	199	109-216	U/I
ALP	396	183	299	107-330	U/I
γ-GTP	19	23	24	<45	U/I
BUN	17	13		8.0-20.0	mg/dl
Creatinine	0.62	0.57		0.38-0.90	mg/dl
Na	138	132	133	136-148	mmol/l
K	5.1	4.9	4.2	3.6-5.0	mmol/l
Cl	102	101	99	96-108	mmol/l
CRP	0.17	3.13	0.39	0.00-0.14	mg/dl
AFP	18594			<10	(ng/ml)
PIVKA-II	272			<40	(mAU/ml)

**Figure 1:**
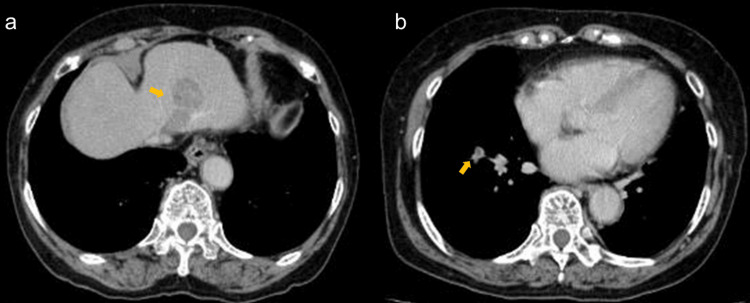
Radiologic findings before surgery. (a) Enhanced computed tomography showed heterogenous 28 mm and 17 mm low-density areas as suspected hepatic tumors and hepatic vein tumor embolus. (b) Enhanced computed tomography showed pulmonary thromboembolism in a right lung field (arrow).

Contrast-enhanced computed tomography (CT) scan findings showed that 28 mm and 17 mm diameter, a round-shaped tumor enhanced heterogeneously with infiltration in the left hepatic vein (Figure 1a). Thromboembolism in the peripheral pulmonary artery was detected, but deep venous thromboembolism was not (Figure 1b).

**Figure 2:**
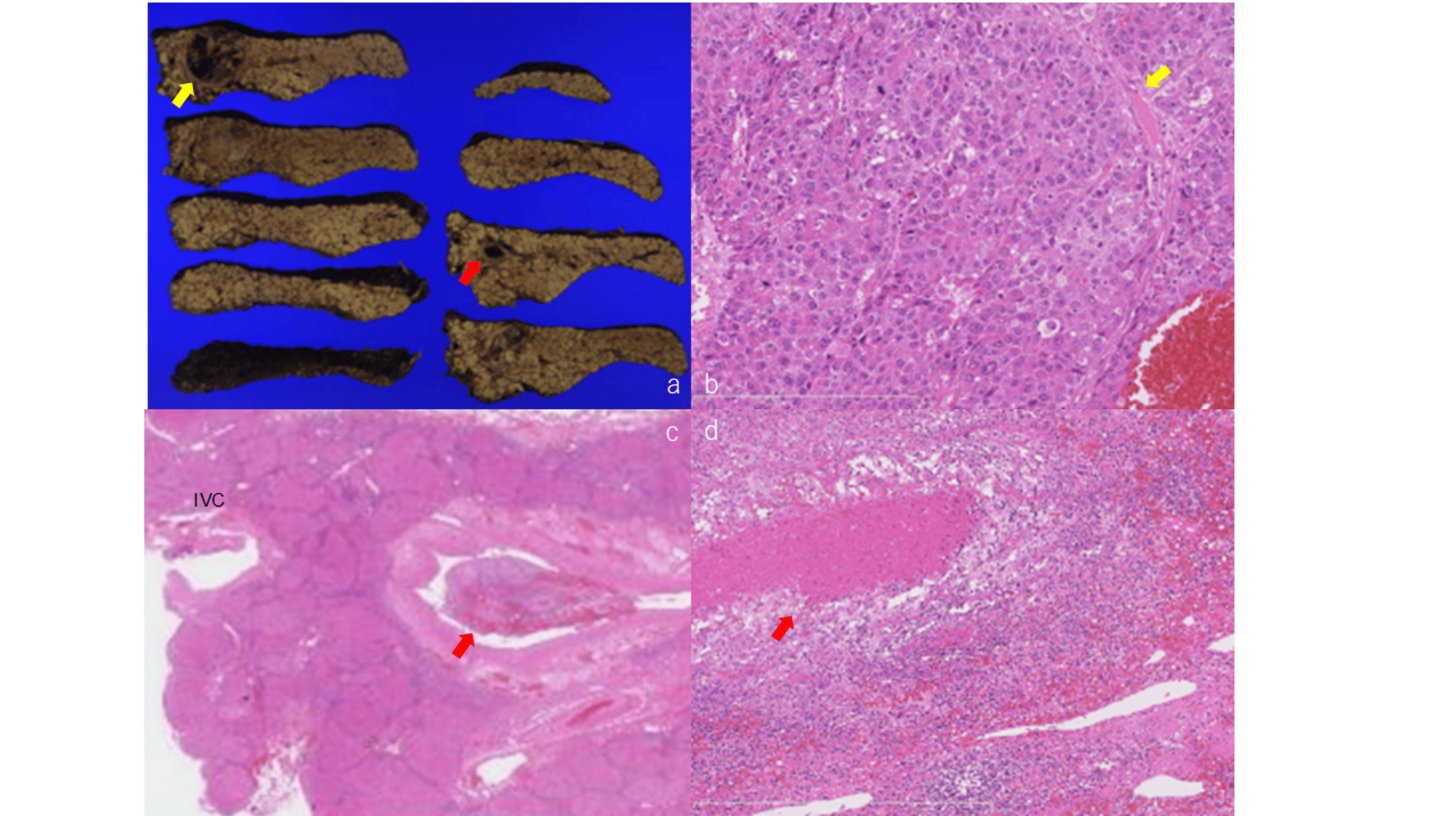
Histopathological examination of the resected specimen. (a) Macroscopic findings show a rough liver surface, suggesting cirrhosis, encapsulated lesion with confluent multinodular appearance (30 × 26 × 25 mm) (yellow arrow), and portal vein thrombosis (red arrow) near the inferior vena cava, (b) Proliferation of the trabecular pattern or pseudo glandular pattern (yellow arrow) (Hematoxylin and eosin stain, magnification ×100), (c) Portal vein embolus (red arrow), which preoperative examinations determined, is not a tumor embolus, but an organized thrombus (×20), (d) Portal vein embolus (red arrow) (×100) IVC: Inferior vena cava

She was diagnosed with liver cancer with venous tumor embolus and pulmonary thromboembolism. The preoperative heparin anticoagulation for the treatment of pulmonary thromboembolism was started. She underwent lateral segmentectomy along with resection of venous tumor embolus. Pathological findings showed that the tumor was moderate to well-differentiated adenocarcinoma invading the capsule along with liver cirrhosis (Figure 2a). Tumor embolization that was suspected preoperatively was an organizing thrombus (Figure 2b).

**Figure 3:**
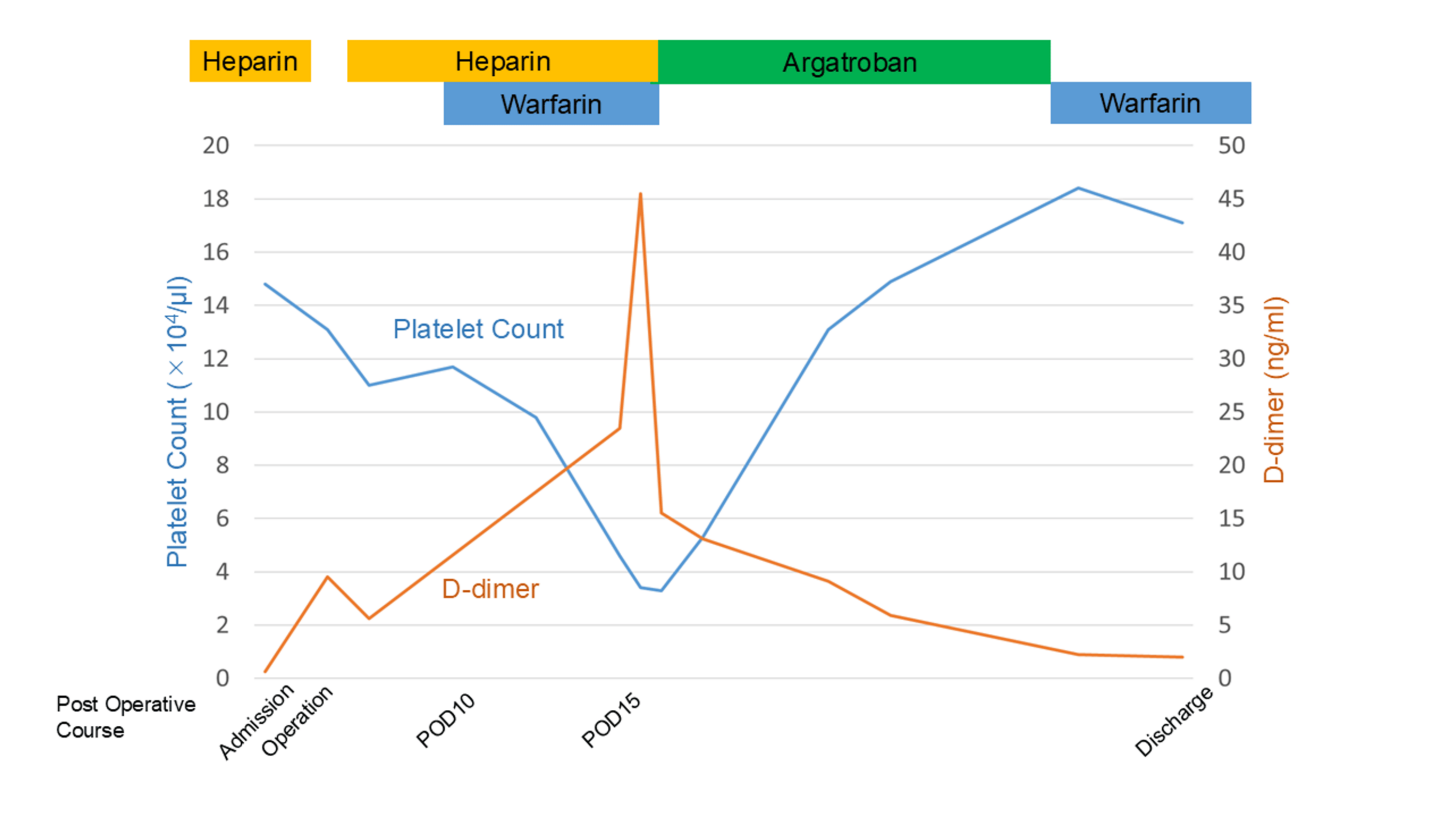
Changes in laboratory findings. POD: Postoperative day The image was created by the authors of this article.

**Figure 4:**
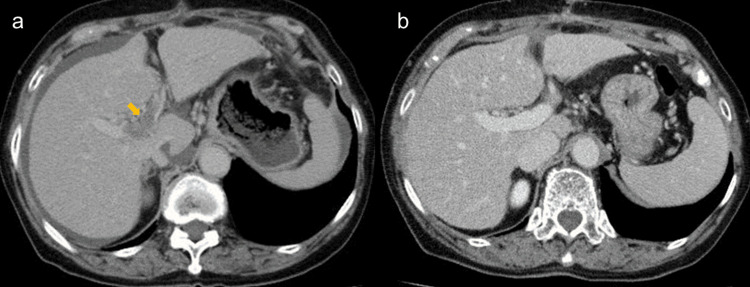
Radiologic findings before and after anticoagulation therapy. (a) Enhanced computed tomography showed portal vein thrombosis (arrow) and ascites on postoperative day 15. (b) Enhanced computed tomography showed complete remission of thrombosis on postoperative day 25.

Postoperatively, heparin was administered again for the routine prophylaxis of deep venous thromboembolism on postoperative day 3. On postoperative day 10, warfarin was administered with heparin. On postoperative day 15, sudden onset of platelet decline (4.6 × 104/μl) and the activity of antithrombin III decline (49.3%) and D-dimer increase (23.5 ng/ml) were pointed out (Table 1). In a laboratory test, there were no liver functional changes. The 4Ts score was 7. HIT was suspected based on the clinical findings. On physical examination, there were no symptoms. Enhanced CT scan findings showed portal vein thrombus (Figure 4a). The anti-HIT antibody was examined and increased (3.3 U/ml). She was diagnosed with HIT. Heparin administration was stopped, and she was treated with argatroban. The platelet level increased to the normal range, and the D-dimer level decreased gradually (Figure 3). On postoperative day 25, a contrast-enhanced CT scan showed complete remission of portal vein thrombus (Figure 4b).

Thereafter, warfarin was started. She was discharged on postoperative day 28.

## Discussion

Postoperative portal vein thrombosis is a serious disease that induces a delay in the improvement of liver damage after hepatectomy and provokes portal vein hypertension in a perioperative period. Hepatocellular carcinoma is often accompanied by liver cirrhosis, which is a well-known risk factor of portal vein thrombosis, however, portal vein thrombosis developed with HIT may be rare in patients with hepatocellular carcinoma with hepatectomy. HIT is associated with hemorrhage as well as the development of systemic thrombosis, which usually involves skin lesions of deep vein thrombosis. To our knowledge, this is the first report of HIT involving portal vein thrombosis formation after hepatectomy.

There may be few reports on the incidence of HIT after hepatectomy because patients with hepatocellular carcinoma are often associated with coagulopathy due to cirrhosis and may be administered anticoagulant therapy in the perioperative period [4]. Moreover, there are few reports about the incidence of HIT with portal vein thrombosis [5]. Postoperative portal vein thrombosis is a lethal complication and is diagnosed in 4.1% of liver resection [6], 19％ of open splenectomy, 55％ of laparoscopic splenectomy [7], 3.5% of pancreatectomy [8], 6％ of colectomy for inflammatory bowel disease [2]. James et al. in 2009 described 18 cases of portovenous thrombosis following laparoscopic cases, with the conclusion that the thrombosis was likely secondary to venous stasis from increased intraabdominal pressure from insufflation, as well as a systemic thrombophilic state [9]. For the diagnosis of portal vein thrombosis, a CT scan may play an important role [5].

We used D-dimer in the follow-up of portal vein thrombosis because D-dimer is a degradation product after fibrin formation, suggesting the presence of a preceding thrombus, which is useful for the maker and follow-up of deep vein thrombosis. In this case report, D-dimer was elevated during the process of thrombus formation and was relieved by thrombolysis after administration of argatroban. D-dimer may be useful in the follow-up of portal vein thrombosis.

To establish the diagnosis of HIT, the 4T’s score is often used. Heparin forms complexes with platelet factor 4 and the Immunoglobulin G antibody for them, referred to as the HIT antibody [10-12]. The HIT antibody activates platelets through the bond to heparin and platelet factor 4 complexes with a resulting decrease in the platelet count by wasting [13]. The HIT antibody is also reported to activate monocytes and neutrophils (pan-cellular activation), causing arterial thrombosis (70%) and venous thrombosis (30%) by endothelial cell injury [14].

A recent analysis of the Nationwide Inpatient Sample in the United States found a 0.63% incidence of HIT in patients after cardiac surgery with cardiopulmonary bypass, consistent with the incidence reported in prospective studies [15-17]. According to the Ministry of Health, Labor, and Welfare in Japan, HIT happens with heparin in 0 to 3.5% of internal medicine disorders, in 1 to 4％ of surgical diseases, and it is reported that HIT happens in 1 to 4％ of traumatic diseases [18].

Typically, thrombocytopenia with HIT occurs five to ten days after the exposure of heparin, produces various thromboembolism, and results in a 10％ hospital mortality rate [15]. HIT antibody is transient and is maintained for 40 to 100 days [10-12]. It is often reported that the concentration of heparin for preventive anticoagulant therapy induces HIT, but heparin flush to wash drip line also induces HIT.

In this case, portal vein thrombosis could be treated safely with argatroban despite the postoperative risk of postoperative hemorrhage without postoperative bleeding. Taguchi et al. reported the usage of argatroban for transplant patients with a history of manifested HIT [19]. For the treatment of HIT, another anticoagulation therapy is considered instead of heparin. The direct thrombin inhibitor argatroban has been approved for prophylaxis and treatment of HIT in the United States and Europe. The direct thrombin inhibitor bivalirudin is often used “off-label” during cardiovascular surgery and in the intensive care setting, where it has been extensively studied and is recommended in guidelines [20].

Although it may be difficult to explain the etiology of the development of portal vein thrombosis and HIT, this patient has several risk factors for portal vein thrombosis formation: one is HIT, the second is viral hepatitis, and the third is post hepatic resection in this patient.

## Conclusions

We reported a rare case of HIT and portal vein thrombosis, which was successfully treated by argatroban without serious bleeding complications after hepatectomy for hepatocellular carcinoma. In gastrointestinal surgery, especially after hepatic resection, HIT should be considered in the development of portal vein thrombosis in case of rapid decrease of platelet and increase of D-dimer. Intraabdominal contrast-enhanced CT may be useful for detecting portal vein thrombosis with or without HIT. The direct thrombin inhibitor argatroban may be useful for the treatment of HIT and portal vein thrombosis.
